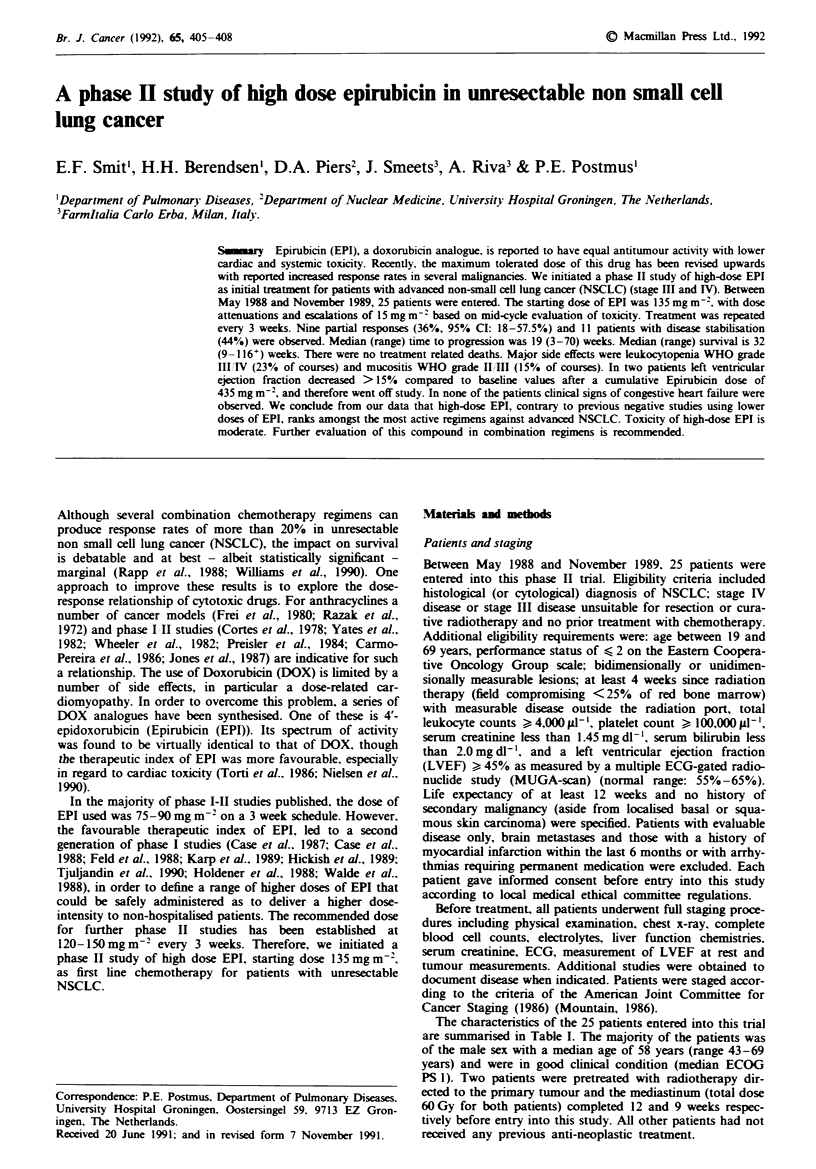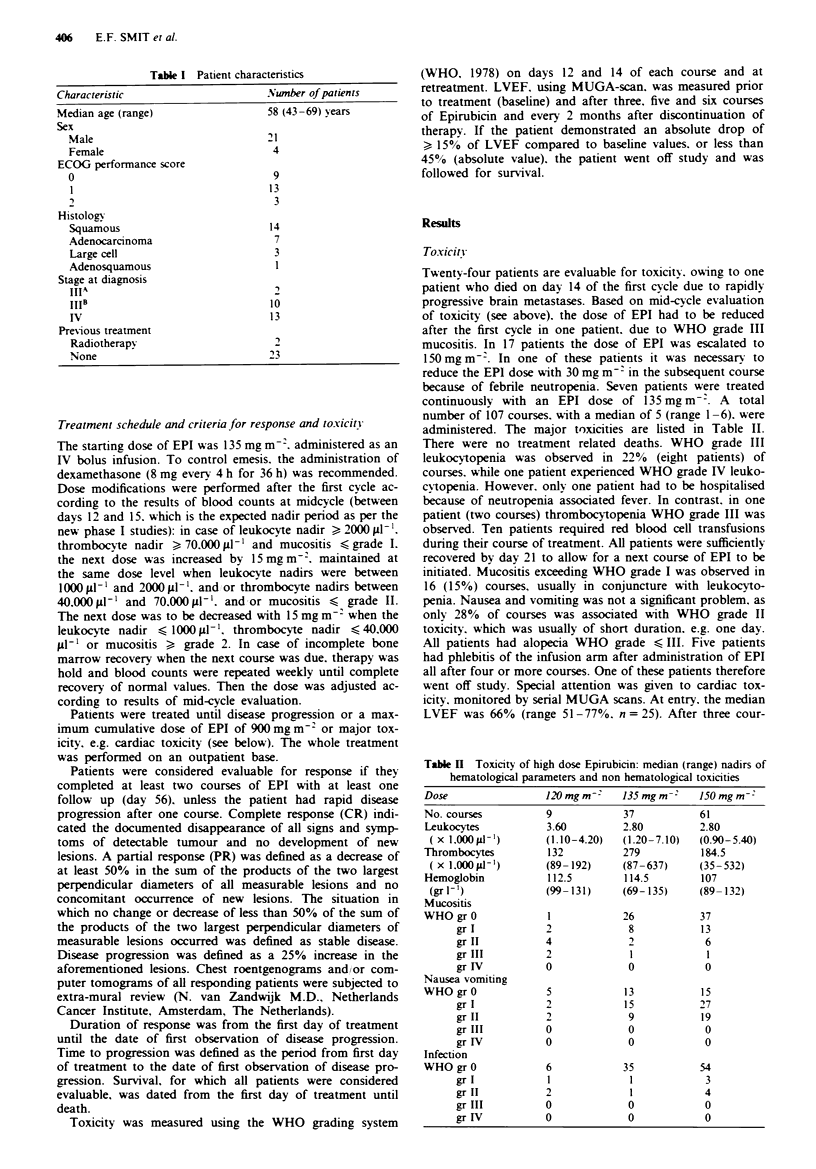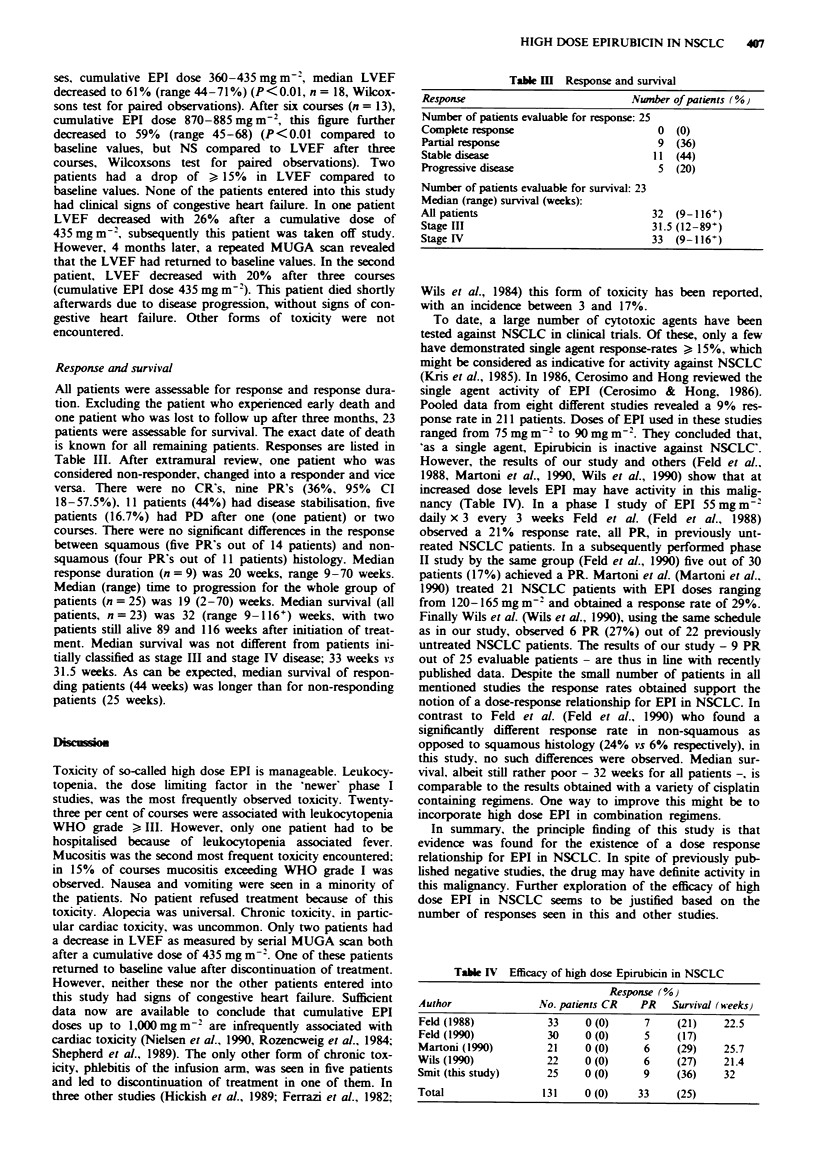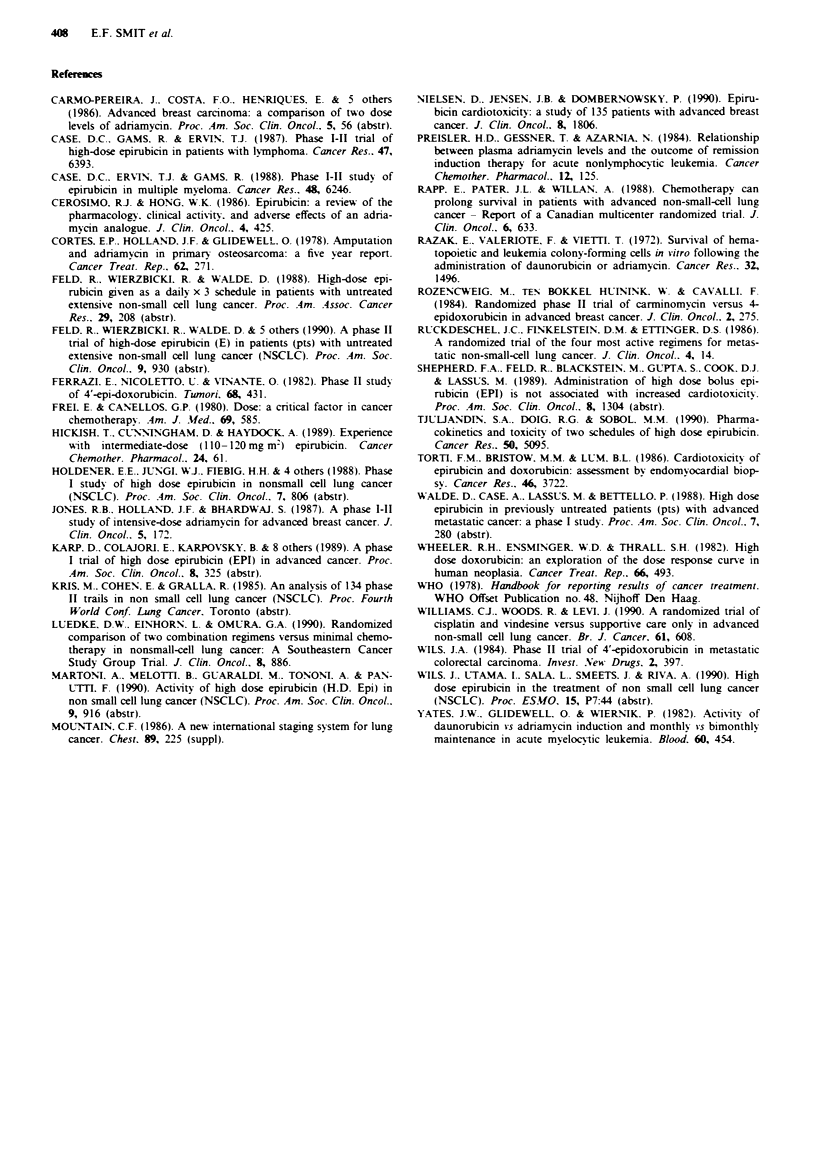# A phase II study of high dose epirubicin in unresectable non small cell lung cancer.

**DOI:** 10.1038/bjc.1992.82

**Published:** 1992-03

**Authors:** E. F. Smit, H. H. Berendsen, D. A. Piers, J. Smeets, A. Riva, P. E. Postmus

**Affiliations:** Department of Pulmonary Diseases, University Hospital Groningen, The Netherlands.

## Abstract

Epirubicin (EPI), a doxorubicin analogue, is reported to have equal antitumour activity with lower cardiac and systemic toxicity. Recently, the maximum tolerated dose of this drug has been revised upwards with reported increased response rates in several malignancies. We initiated a phase II study of high-dose EPI as initial treatment for patients with advanced non-small cell lung cancer (NSCLC) (stage III and IV). Between May 1988 and November 1989, 25 patients were entered. The starting dose of EPI was 135 mg m-2, with dose attenuations and escalations of 15 mg m-2 based on mid-cycle evaluation of toxicity. Treatment was repeated every 3 weeks. Nine partial responses (36%, 95% CI: 18-57.5%) and 11 patients with disease stabilisation (44%) were observed. Median (range) time to progression was 19 (3-70) weeks. Median (range) survival is 32 (9-116+) weeks. There were no treatment related deaths. Major side effects were leukocytopenia WHO grade III/IV (23% of courses) and mucositis WHO grade II/III (15% of courses). In two patients left ventricular ejection fraction decreased greater than 15% compared to baseline values after a cumulative Epirubicin dose of 435 mg m-2, and therefore went off study. In none of the patients clinical signs of congestive heart failure were observed. We conclude from our data that high-dose EPI, contrary to previous negative studies using lower doses of EPI, ranks amongst the most active regimens against advanced NSCLC. Toxicity of high-dose EPI is moderate. Further evaluation of this compound in combination regimens is recommended.


Br. J. Cancer (1992), 65, 405–408 © Macmillan Press Ltd., 1992

### A phase II study of high dose epirubicin in unresectable non small cell lung cancer

E.F. Smit[1], H.H. Berendsen[1], D.A. Piers[2], J. Smeets[3], A. Riva[3] & P.E. Postmus[1]

[1]*Department of Pulmonary Diseases,* [2]*Department of Nuclear Medicine, University Hospital Groningen, The Netherlands,* [3]*FarmItalia Carlo Erba, Milan, Italy.*

**Summary** Epirubicin (EPI), a doxorubicin analogue, is reported to have equal antitumour activity with lower cardiac and systemic toxicity. Recently, the maximum tolerated dose of this drug has been revised upwards with reported increased response rates in several malignancies. We initiated a phase II study of high-dose EPI as initial treatment for patients with advanced non-small cell lung cancer (NSCLC) (stage III and IV). Between May 1988 and November 1989, 25 patients were entered. The starting dose of EPI was 135 mg m$^{-2}$, with dose attenuations and escalations of 15 mg m$^{-2}$ based on mid-cycle evaluation of toxicity. Treatment was repeated every 3 weeks. Nine partial responses (36%, 95% CI: 18–57.5%) and 11 patients with disease stabilisation (44%) were observed. Median (range) time to progression was 19 (3–70) weeks. Median (range) survival is 32 (9–116$^{+}$) weeks. There were no treatment related deaths. Major side effects were leukocytopenia WHO grade III/IV (23% of courses) and mucositis WHO grade II/III (15% of courses). In two patients left ventricular ejection fraction decreased >15% compared to baseline values after a cumulative Epirubicin dose of 435 mg m$^{-2}$, and therefore went off study. In none of the patients clinical signs of congestive heart failure were observed. We conclude from our data that high-dose EPI, contrary to previous negative studies using lower doses of EPI, ranks amongst the most active regimens against advanced NSCLC. Toxicity of high-dose EPI is moderate. Further evaluation of this compound in combination regimens is recommended.

Although several combination chemotherapy regimens can produce response rates of more than 20% in unresectable non small cell lung cancer (NSCLC), the impact on survival is debatable and at best – albeit statistically significant – marginal (Rapp *et al.*, 1988; Williams *et al.*, 1990). One approach to improve these results is to explore the dose-response relationship of cytotoxic drugs. For anthracyclines a number of cancer models (Frei *et al.*, 1980; Razak *et al.*, 1972) and phase I/II studies (Cortes *et al.*, 1978; Yates *et al.*, 1982; Wheeler *et al.*, 1982; Preisler *et al.*, 1984; Carmo-Pereira *et al.*, 1986; Jones *et al.*, 1987) are indicative for such a relationship. The use of Doxorubicin (DOX) is limited by a number of side effects, in particular a dose-related cardiomyopathy. In order to overcome this problem, a series of DOX analogues have been synthesised. One of these is 4'-epidoxorubicin (Epirubicin (EPI)). Its spectrum of activity was found to be virtually identical to that of DOX, though the therapeutic index of EPI was more favourable, especially in regard to cardiac toxicity (Torti *et al.*, 1986; Nielsen *et al.*, 1990).

In the majority of phase I-II studies published, the dose of EPI used was 75–90 mg m$^{-2}$ on a 3 week schedule. However, the favourable therapeutic index of EPI, led to a second generation of phase I studies (Case *et al.*, 1987; Case *et al.*, 1988; Feld *et al.*, 1988; Karp *et al.*, 1989; Hickish *et al.*, 1989; Tjuljandin *et al.*, 1990; Holdener *et al.*, 1988; Walde *et al.*, 1988), in order to define a range of higher doses of EPI that could be safely administered as to deliver a higher dose-intensity to non-hospitalised patients. The recommended dose for further phase II studies has been established at 120–150 mg m$^{-2}$ every 3 weeks. Therefore, we initiated a phase II study of high dose EPI, starting dose 135 mg m$^{-2}$, as first line chemotherapy for patients with unresectable NSCLC.

Correspondence: P.E. Postmus, Department of Pulmonary Diseases, University Hospital Groningen, Oostersingel 59, 9713 EZ Groningen, The Netherlands.
Received 20 June 1991; and in revised form 7 November 1991.

### Materials and methods

### *Patients and staging*

Between May 1988 and November 1989, 25 patients were entered into this phase II trial. Eligibility criteria included histological (or cytological) diagnosis of NSCLC; stage IV disease or stage III disease unsuitable for resection or curative radiotherapy and no prior treatment with chemotherapy. Additional eligibility requirements were: age between 19 and 69 years, performance status of ⩽ 2 on the Eastern Cooperative Oncology Group scale; bidimensionally or unidimensionally measurable lesions; at least 4 weeks since radiation therapy (field compromising <25% of red bone marrow) with measurable disease outside the radiation port, total leukocyte counts ⩾ 4,000 μl$^{-1}$, platelet count ⩾ 100,000 μl$^{-1}$, serum creatinine less than 1.45 mg dl$^{-1}$, serum bilirubin less than 2.0 mg dl$^{-1}$, and a left ventricular ejection fraction (LVEF) ⩾ 45% as measured by a multiple ECG-gated radionuclide study (MUGA-scan) (normal range: 55%–65%). Life expectancy of at least 12 weeks and no history of secondary malignancy (aside from localised basal or squamous skin carcinoma) were specified. Patients with evaluable disease only, brain metastases and those with a history of myocardial infarction within the last 6 months or with arrhythmias requiring permanent medication were excluded. Each patient gave informed consent before entry into this study according to local medical ethical committee regulations.

Before treatment, all patients underwent full staging procedures including physical examination, chest x-ray, complete blood cell counts, electrolytes, liver function chemistries, serum creatinine, ECG, measurement of LVEF at rest and tumour measurements. Additional studies were obtained to document disease when indicated. Patients were staged according to the criteria of the American Joint Committee for Cancer Staging (1986) (Mountain, 1986).

The characteristics of the 25 patients entered into this trial are summarised in Table I. The majority of the patients was of the male sex with a median age of 58 years (range 43–69 years) and were in good clinical condition (median ECOG PS 1). Two patients were pretreated with radiotherapy directed to the primary tumour and the mediastinum (total dose 60 Gy for both patients) completed 12 and 9 weeks respectively before entry into this study. All other patients had not received any previous anti-neoplastic treatment.

**Table I** Patient characteristics

| *Characteristic* | *Number of patients* |
|---|---|
| Median age (range) | 58 (43–69) years |
| Sex | |
| Male | 21 |
| Female | 4 |
| ECOG performance score | |
| 0 | 9 |
| 1 | 13 |
| 2 | 3 |
| Histology | |
| Squamous | 14 |
| Adenocarcinoma | 7 |
| Large cell | 3 |
| Adenosquamous | 1 |
| Stage at diagnosis | |
| $III^A$ | 2 |
| $III^B$ | 10 |
| IV | 13 |
| Previous treatment | |
| Radiotherapy | 2 |
| None | 23 |

*Treatment schedule and criteria for response and toxicity*

The starting dose of EPI was 135 mg $m^{-2}$, administered as an IV bolus infusion. To control emesis, the administration of dexamethasone (8 mg every 4 h for 36 h) was recommended. Dose modifications were performed after the first cycle according to the results of blood counts at midcycle (between days 12 and 15, which is the expected nadir period as per the new phase I studies): in case of leukocyte nadir $\geqslant 2000\ \mu l^{-1}$, thrombocyte nadir $\geqslant 70.000\ \mu l^{-1}$ and mucositis $\leqslant$ grade I, the next dose was increased by 15 mg $m^{-2}$, maintained at the same dose level when leukocyte nadirs were between 1000 $\mu l^{-1}$ and 2000 $\mu l^{-1}$, and/or thrombocyte nadirs between 40.000 $\mu l^{-1}$ and 70.000 $\mu l^{-1}$, and/or mucositis $\leqslant$ grade II. The next dose was to be decreased with 15 mg $m^{-2}$ when the leukocyte nadir $\leqslant 1000\ \mu l^{-1}$, thrombocyte nadir $\leqslant 40.000\ \mu l^{-1}$ or mucositis $\geqslant$ grade 2. In case of incomplete bone marrow recovery when the next course was due, therapy was hold and blood counts were repeated weekly until complete recovery of normal values. Then the dose was adjusted according to results of mid-cycle evaluation.

Patients were treated until disease progression or a maximum cumulative dose of EPI of 900 mg $m^{-2}$ or major toxicity, e.g. cardiac toxicity (see below). The whole treatment was performed on an outpatient base.

Patients were considered evaluable for response if they completed at least two courses of EPI with at least one follow up (day 56), unless the patient had rapid disease progression after one course. Complete response (CR) indicated the documented disappearance of all signs and symptoms of detectable tumour and no development of new lesions. A partial response (PR) was defined as a decrease of at least 50% in the sum of the products of the two largest perpendicular diameters of all measurable lesions and no concomitant occurrence of new lesions. The situation in which no change or decrease of less than 50% of the sum of the products of the two largest perpendicular diameters of measurable lesions occurred was defined as stable disease. Disease progression was defined as a 25% increase in the aforementioned lesions. Chest roentgenograms and/or computer tomograms of all responding patients were subjected to extra-mural review (N. van Zandwijk M.D., Netherlands Cancer Institute, Amsterdam, The Netherlands).

Duration of response was from the first day of treatment until the date of first observation of disease progression. Time to progression was defined as the period from first day of treatment to the date of first observation of disease progression. Survival, for which all patients were considered evaluable, was dated from the first day of treatment until death.

Toxicity was measured using the WHO grading system (WHO, 1978) on days 12 and 14 of each course and at retreatment. LVEF, using MUGA-scan, was measured prior to treatment (baseline) and after three, five and six courses of Epirubicin and every 2 months after discontinuation of therapy. If the patient demonstrated an absolute drop of $\geqslant 15\%$ of LVEF compared to baseline values, or less than 45% (absolute value), the patient went off study and was followed for survival.

### Results

*Toxicity*

Twenty-four patients are evaluable for toxicity, owing to one patient who died on day 14 of the first cycle due to rapidly progressive brain metastases. Based on mid-cycle evaluation of toxicity (see above), the dose of EPI had to be reduced after the first cycle in one patient, due to WHO grade III mucositis. In 17 patients the dose of EPI was escalated to 150 mg $m^{-2}$. In one of these patients it was necessary to reduce the EPI dose with 30 mg $m^{-2}$ in the subsequent course because of febrile neutropenia. Seven patients were treated continuously with an EPI dose of 135 mg $m^{-2}$. A total number of 107 courses, with a median of 5 (range 1–6), were administered. The major toxicities are listed in Table II. There were no treatment related deaths. WHO grade III leukocytopenia was observed in 22% (eight patients) of courses, while one patient experienced WHO grade IV leukocytopenia. However, only one patient had to be hospitalised because of neutropenia associated fever. In contrast, in one patient (two courses) thrombocytopenia WHO grade III was observed. Ten patients required red blood cell transfusions during their course of treatment. All patients were sufficiently recovered by day 21 to allow for a next course of EPI to be initiated. Mucositis exceeding WHO grade I was observed in 16 (15%) courses, usually in conjuncture with leukocytopenia. Nausea and vomiting was not a significant problem, as only 28% of courses was associated with WHO grade II toxicity, which was usually of short duration, e.g. one day. All patients had alopecia WHO grade $\leqslant$ III. Five patients had phlebitis of the infusion arm after administration of EPI all after four or more courses. One of these patients therefore went off study. Special attention was given to cardiac toxicity, monitored by serial MUGA scans. At entry, the median LVEF was 66% (range 51–77%, $n = 25$). After three cour-

**Table II** Toxicity of high dose Epirubicin: median (range) nadirs of hematological parameters and non hematological toxicities

| *Dose* | *120 mg $m^{-2}$* | *135 mg $m^{-2}$* | *150 mg $m^{-2}$* |
|---|---|---|---|
| No. courses | 9 | 37 | 61 |
| Leukocytes | 3.60 | 2.80 | 2.80 |
| ( × 1,000 $\mu l^{-1}$) | (1.10–4.20) | (1.20–7.10) | (0.90–5.40) |
| Thrombocytes | 132 | 279 | 184.5 |
| ( × 1,000 $\mu l^{-1}$) | (89–192) | (87–637) | (35–532) |
| Hemoglobin | 112.5 | 114.5 | 107 |
| (gr $l^{-1}$) | (99–131) | (69–135) | (89–132) |
| Mucositis | | | |
| WHO gr 0 | 1 | 26 | 37 |
| gr I | 2 | 8 | 13 |
| gr II | 4 | 2 | 6 |
| gr III | 2 | 1 | 1 |
| gr IV | 0 | 0 | 0 |
| Nausea vomiting | | | |
| WHO gr 0 | 5 | 13 | 15 |
| gr I | 2 | 15 | 27 |
| gr II | 2 | 9 | 19 |
| gr III | 0 | 0 | 0 |
| gr IV | 0 | 0 | 0 |
| Infection | | | |
| WHO gr 0 | 6 | 35 | 54 |
| gr I | 1 | 1 | 3 |
| gr II | 2 | 1 | 4 |
| gr III | 0 | 0 | 0 |
| gr IV | 0 | 0 | 0 |

ses, cumulative EPI dose 360–435 mg m$^{-2}$, median LVEF decreased to 61% (range 44–71%) ($P<0.01$, $n = 18$, Wilcoxsons test for paired observations). After six courses ($n = 13$), cumulative EPI dose 870–885 mg m$^{-2}$, this figure further decreased to 59% (range 45–68) ($P<0.01$ compared to baseline values, but NS compared to LVEF after three courses, Wilcoxsons test for paired observations). Two patients had a drop of $\geqslant 15\%$ in LVEF compared to baseline values. None of the patients entered into this study had clinical signs of congestive heart failure. In one patient LVEF decreased with 26% after a cumulative dose of 435 mg m$^{-2}$, subsequently this patient was taken off study. However, 4 months later, a repeated MUGA scan revealed that the LVEF had returned to baseline values. In the second patient, LVEF decreased with 20% after three courses (cumulative EPI dose 435 mg m$^{-2}$). This patient died shortly afterwards due to disease progression, without signs of congestive heart failure. Other forms of toxicity were not encountered.

*Response and survival*

All patients were assessable for response and response duration. Excluding the patient who experienced early death and one patient who was lost to follow up after three months, 23 patients were assessable for survival. The exact date of death is known for all remaining patients. Responses are listed in Table III. After extramural review, one patient who was considered non-responder, changed into a responder and vice versa. There were no CR's, nine PR's (36%, 95% CI 18–57.5%), 11 patients (44%) had disease stabilisation, five patients (16.7%) had PD after one (one patient) or two courses. There were no significant differences in the response between squamous (five PR's out of 14 patients) and non-squamous (four PR's out of 11 patients) histology. Median response duration ($n = 9$) was 20 weeks, range 9–70 weeks. Median (range) time to progression for the whole group of patients ($n = 25$) was 19 (2–70) weeks. Median survival (all patients, $n = 23$) was 32 (range 9–116$^{+}$) weeks, with two patients still alive 89 and 116 weeks after initiation of treatment. Median survival was not different from patients initially classified as stage III and stage IV disease; 33 weeks *vs* 31.5 weeks. As can be expected, median survival of responding patients (44 weeks) was longer than for non-responding patients (25 weeks).

**Discussion**

Toxicity of so-called high dose EPI is manageable. Leukocytopenia, the dose limiting factor in the 'newer' phase I studies, was the most frequently observed toxicity. Twenty-three per cent of courses were associated with leukocytopenia WHO grade $\geqslant$ III. However, only one patient had to be hospitalised because of leukocytopenia associated fever. Mucositis was the second most frequent toxicity encountered; in 15% of courses mucositis exceeding WHO grade I was observed. Nausea and vomiting were seen in a minority of the patients. No patient refused treatment because of this toxicity. Alopecia was universal. Chronic toxicity, in particular cardiac toxicity, was uncommon. Only two patients had a decrease in LVEF as measured by serial MUGA scan both after a cumulative dose of 435 mg m$^{-2}$. One of these patients returned to baseline value after discontinuation of treatment. However, neither these nor the other patients entered into this study had signs of congestive heart failure. Sufficient data now are available to conclude that cumulative EPI doses up to 1,000 mg m$^{-2}$ are infrequently associated with cardiac toxicity (Nielsen *et al.*, 1990, Rozencweig *et al.*, 1984; Shepherd *et al.*, 1989). The only other form of chronic toxicity, phlebitis of the infusion arm, was seen in five patients and led to discontinuation of treatment in one of them. In three other studies (Hickish *et al.*, 1989; Ferrazi *et al.*, 1982; Wils *et al.*, 1984) this form of toxicity has been reported, with an incidence between 3 and 17%.

**Table III** Response and survival

| *Response* | *Number of patients (%)* |
|---|---|
| Number of patients evaluable for response: 25 | |
| Complete response | 0 (0) |
| Partial response | 9 (36) |
| Stable disease | 11 (44) |
| Progressive disease | 5 (20) |
| Number of patients evaluable for survival: 23 | |
| Median (range) survival (weeks): | |
| All patients | 32 (9–116$^{+}$) |
| Stage III | 31.5 (12–89$^{+}$) |
| Stage IV | 33 (9–116$^{+}$) |

To date, a large number of cytotoxic agents have been tested against NSCLC in clinical trials. Of these, only a few have demonstrated single agent response-rates $\geqslant 15\%$, which might be considered as indicative for activity against NSCLC (Kris *et al.*, 1985). In 1986, Cerosimo and Hong reviewed the single agent activity of EPI (Cerosimo & Hong, 1986). Pooled data from eight different studies revealed a 9% response rate in 211 patients. Doses of EPI used in these studies ranged from 75 mg m$^{-2}$ to 90 mg m$^{-2}$. They concluded that, 'as a single agent, Epirubicin is inactive against NSCLC'. However, the results of our study and others (Feld *et al.*, 1988, Martoni *et al.*, 1990, Wils *et al.*, 1990) show that at increased dose levels EPI may have activity in this malignancy (Table IV). In a phase I study of EPI 55 mg m$^{-2}$ daily $\times$ 3 every 3 weeks Feld *et al.* (Feld *et al.*, 1988) observed a 21% response rate, all PR, in previously untreated NSCLC patients. In a subsequently performed phase II study by the same group (Feld *et al.*, 1990) five out of 30 patients (17%) achieved a PR. Martoni *et al.* (Martoni *et al.*, 1990) treated 21 NSCLC patients with EPI doses ranging from 120–165 mg m$^{-2}$ and obtained a response rate of 29%. Finally Wils *et al.* (Wils *et al.*, 1990), using the same schedule as in our study, observed 6 PR (27%) out of 22 previously untreated NSCLC patients. The results of our study – 9 PR out of 25 evaluable patients – are thus in line with recently published data. Despite the small number of patients in all mentioned studies the response rates obtained support the notion of a dose-response relationship for EPI in NSCLC. In contrast to Feld *et al.* (Feld *et al.*, 1990) who found a significantly different response rate in non-squamous as opposed to squamous histology (24% *vs* 6% respectively), in this study, no such differences were observed. Median survival, albeit still rather poor – 32 weeks for all patients –, is comparable to the results obtained with a variety of cisplatin containing regimens. One way to improve this might be to incorporate high dose EPI in combination regimens.

In summary, the principle finding of this study is that evidence was found for the existence of a dose response relationship for EPI in NSCLC. In spite of previously published negative studies, the drug may have definite activity in this malignancy. Further exploration of the efficacy of high dose EPI in NSCLC seems to be justified based on the number of responses seen in this and other studies.

**Table IV** Efficacy of high dose Epirubicin in NSCLC

| *Author* | *No. patients* | *Response (%)* *CR* | *PR* | | *Survival (weeks)* |
|---|---|---|---|---|---|
| Feld (1988) | 33 | 0 (0) | 7 | (21) | 22.5 |
| Feld (1990) | 30 | 0 (0) | 5 | (17) | |
| Martoni (1990) | 21 | 0 (0) | 6 | (29) | 25.7 |
| Wils (1990) | 22 | 0 (0) | 6 | (27) | 21.4 |
| Smit (this study) | 25 | 0 (0) | 9 | (36) | 32 |
| Total | 131 | 0 (0) | 33 | (25) | |

**References**

CARMO-PEREIRA, J., COSTA, F.O., HENRIQUES, E. & 5 others (1986). Advanced breast carcinoma: a comparison of two dose levels of adriamycin. *Proc. Am. Soc. Clin. Oncol.*, **5**, 56 (abstr).

CASE, D.C., GAMS, R. & ERVIN, T.J. (1987). Phase I-II trial of high-dose epirubicin in patients with lymphoma. *Cancer Res.*, **47**, 6393.

CASE, D.C., ERVIN, T.J. & GAMS, R. (1988). Phase I-II study of epirubicin in multiple myeloma. *Cancer Res.*, **48**, 6246.

CEROSIMO, R.J. & HONG, W.K. (1986). Epirubicin: a review of the pharmacology, clinical activity, and adverse effects of an adriamycin analogue. *J. Clin. Oncol.*, **4**, 425.

CORTES, E.P., HOLLAND, J.F. & GLIDEWELL, O. (1978). Amputation and adriamycin in primary osteosarcoma: a five year report. *Cancer Treat. Rep.*, **62**, 271.

FELD, R., WIERZBICKI, R. & WALDE, D. (1988). High-dose epirubicin given as a daily × 3 schedule in patients with untreated extensive non-small cell lung cancer. *Proc. Am. Assoc. Cancer Res.*, **29**, 208 (abstr).

FELD, R., WIERZBICKI, R., WALDE, D. & 5 others (1990). A phase II trial of high-dose epirubicin (E) in patients (pts) with untreated extensive non-small cell lung cancer (NSCLC). *Proc. Am. Soc. Clin. Oncol.*, **9**, 930 (abstr).

FERRAZI, E., NICOLETTO, U. & VINANTE, O. (1982). Phase II study of 4'-epi-doxorubicin. *Tumori*, **68**, 431.

FREI, E. & CANELLOS, G.P. (1980). Dose: a critical factor in cancer chemotherapy. *Am. J. Med.*, **69**, 585.

HICKISH, T., CUNNINGHAM, D. & HAYDOCK, A. (1989). Experience with intermediate-dose (110–120 mg $m^2$) epirubicin. *Cancer Chemother. Pharmacol.*, **24**, 61.

HOLDENER, E.E., JUNGI, W.J., FIEBIG, H.H. & 4 others (1988). Phase I study of high dose epirubicin in nonsmall cell lung cancer (NSCLC). *Proc. Am. Soc. Clin. Oncol.*, **7**, 806 (abstr).

JONES, R.B., HOLLAND, J.F. & BHARDWAJ, S. (1987). A phase I-II study of intensive-dose adriamycin for advanced breast cancer. *J. Clin. Oncol.*, **5**, 172.

KARP, D., COLAJORI, E., KARPOVSKY, B. & 8 others (1989). A phase I trial of high dose epirubicin (EPI) in advanced cancer. *Proc. Am. Soc. Clin. Oncol.*, **8**, 325 (abstr).

KRIS, M., COHEN, E. & GRALLA, R. (1985). An analysis of 134 phase II trails in non small cell lung cancer (NSCLC). *Proc. Fourth World Conf. Lung Cancer*, Toronto (abstr).

LUEDKE, D.W., EINHORN, L. & OMURA, G.A. (1990). Randomized comparison of two combination regimens versus minimal chemotherapy in nonsmall-cell lung cancer: A Southeastern Cancer Study Group Trial. *J. Clin. Oncol.*, **8**, 886.

MARTONI, A., MELOTTI, B., GUARALDI, M., TONONI, A. & PANUTTI, F. (1990). Activity of high dose epirubicin (H.D. Epi) in non small cell lung cancer (NSCLC). *Proc. Am. Soc. Clin. Oncol.*, **9**, 916 (abstr).

MOUNTAIN, C.F. (1986). A new international staging system for lung cancer. *Chest*, **89**, 225 (suppl).

NIELSEN, D., JENSEN, J.B. & DOMBERNOWSKY, P. (1990). Epirubicin cardiotoxicity: a study of 135 patients with advanced breast cancer. *J. Clin. Oncol.*, **8**, 1806.

PREISLER, H.D., GESSNER, T. & AZARNIA, N. (1984). Relationship between plasma adriamycin levels and the outcome of remission induction therapy for acute nonlymphocytic leukemia. *Cancer Chemother. Pharmacol.*, **12**, 125.

RAPP, E., PATER, J.L. & WILLAN, A. (1988). Chemotherapy can prolong survival in patients with advanced non-small-cell lung cancer – Report of a Canadian multicenter randomized trial. *J. Clin. Oncol.*, **6**, 633.

RAZAK, E., VALERIOTE, F. & VIETTI, T. (1972). Survival of hematopoietic and leukemia colony-forming cells *in vitro* following the administration of daunorubicin or adriamycin. *Cancer Res.*, **32**, 1496.

ROZENCWEIG, M., TEN BOKKEL HUININK, W. & CAVALLI, F. (1984). Randomized phase II trial of carminomycin versus 4-epidoxorubicin in advanced breast cancer. *J. Clin. Oncol.*, **2**, 275.

RUCKDESCHEL, J.C., FINKELSTEIN, D.M. & ETTINGER, D.S. (1986). A randomized trial of the four most active regimens for metastatic non-small-cell lung cancer. *J. Clin. Oncol.*, **4**, 14.

SHEPHERD, F.A., FELD, R., BLACKSTEIN, M., GUPTA, S., COOK, D.J. & LASSUS, M. (1989). Administration of high dose bolus epirubicin (EPI) is not associated with increased cardiotoxicity. *Proc. Am. Soc. Clin. Oncol.*, **8**, 1304 (abstr).

TJULJANDIN, S.A., DOIG, R.G. & SOBOL, M.M. (1990). Pharmacokinetics and toxicity of two schedules of high dose epirubicin. *Cancer Res.*, **50**, 5095.

TORTI, F.M., BRISTOW, M.M. & LUM, B.L. (1986). Cardiotoxicity of epirubicin and doxorubicin: assessment by endomyocardial biopsy. *Cancer Res.*, **46**, 3722.

WALDE, D., CASE, A., LASSUS, M. & BETTELLO, P. (1988). High dose epirubicin in previously untreated patients (pts) with advanced metastatic cancer: a phase I study. *Proc. Am. Soc. Clin. Oncol.*, **7**, 280 (abstr).

WHEELER, R.H., ENSMINGER, W.D. & THRALL, S.H. (1982). High dose doxorubicin: an exploration of the dose response curve in human neoplasia. *Cancer Treat. Rep.*, **66**, 493.

WHO (1978). *Handbook for reporting results of cancer treatment*. WHO Offset Publication no. 48, Nijhoff Den Haag.

WILLIAMS, C.J., WOODS, R. & LEVI, J. (1990. A randomized trial of cisplatin and vindesine versus supportive care only in advanced non-small cell lung cancer. *Br. J. Cancer*, **61**, 608.

WILS, J.A. (1984). Phase II trial of 4'-epidoxorubicin in metastatic colorectal carcinoma. *Invest. New Drugs*, **2**, 397.

WILS, J., UTAMA, I., SALA, L., SMEETS, J. & RIVA, A. (1990). High dose epirubicin in the treatment of non small cell lung cancer (NSCLC). *Proc. ESMO*, **15**, P7:44 (abstr).

YATES, J.W., GLIDEWELL, O. & WIERNIK, P. (1982). Activity of daunorubicin *vs* adriamycin induction and monthly *vs* bimonthly maintenance in acute myelocytic leukemia. *Blood*, **60**, 454.